# Dietary zinc-di-tripeptide enhances zinc relative bioavailability and reduces fecal zinc losses compared to zinc sulfate, without compromising performance of growing pigs

**DOI:** 10.1093/jas/skaf409

**Published:** 2025-11-25

**Authors:** Pedro Silva Careli, Danyel Bueno Dalto, Rayanne Andrade Nunes, Damares De Castro Fidélis Toledo, Marina Mendonça Navarro Penna Barbosa, Paloma Amorim Vaz, Anne-Cecile Jutten, Raquel Tatiane Pereira, Paulo Levi De Oliveira Carvalho, Gabriel Cipriano Rocha, Jansller Luiz Genova

**Affiliations:** Muscle Biology and Nutrigenomics Laboratory, Animal Science Department, Universidade Federal de Viçosa, Viçosa, MG 36570-900, Brazil; Sherbrooke Research and Development Centre, Agriculture and Agri-Food Canada, Sherbrooke, Quebec, J1M 0C8, Canada; Muscle Biology and Nutrigenomics Laboratory, Animal Science Department, Universidade Federal de Viçosa, Viçosa, MG 36570-900, Brazil; Muscle Biology and Nutrigenomics Laboratory, Animal Science Department, Universidade Federal de Viçosa, Viçosa, MG 36570-900, Brazil; Muscle Biology and Nutrigenomics Laboratory, Animal Science Department, Universidade Federal de Viçosa, Viçosa, MG 36570-900, Brazil; Muscle Biology and Nutrigenomics Laboratory, Animal Science Department, Universidade Federal de Viçosa, Viçosa, MG 36570-900, Brazil; Olmix S.A., ZA du Haut du Bois, 56580 Bréhan, France; Olmix S.A., ZA du Haut du Bois, 56580 Bréhan, France; Animal Science Department, Universidade Estadual do Oeste do Paraná, Marechal Cândido Rondon, PR 85960-000, Brazil; Muscle Biology and Nutrigenomics Laboratory, Animal Science Department, Universidade Federal de Viçosa, Viçosa, MG 36570-900, Brazil; Muscle Biology and Nutrigenomics Laboratory, Animal Science Department, Universidade Federal de Viçosa, Viçosa, MG 36570-900, Brazil

**Keywords:** pig performance, serum zinc, tissue zinc retention, zinc bioavailability, zinc digestibility, zinc-peptide

## Abstract

Zinc (Zn) is an essential trace mineral involved in key physiological processes, and organic Zn sources are proposed to have higher bioavailability than inorganic forms. This study aimed to evaluate the bioavailability of a Zn-di-tripeptide (Zn-di-tripep) chelate compared to Zn sulfate (ZnSO_4_) on growth performance, apparent total tract digestibility of Zn and Zn concentrations in feces, serum, liver, and bone in growing pigs. Ninety male pigs (Landrace × Large White, 63 day-old, 25.44 ± 0.302 kg body weight [BW]) were used in a randomized complete block design based on BW with 10 pigs per treatment over a 60-day period. Pigs were allocated to a 2 × 4 + 1 factorial design, composed by 2 Zn sources (Zn–di–tripep vs. ZnSO_4_), 4 levels of dietary Zn (30, 60, 90, and 120 mg/kg diet), and a negative control (no supplemental Zn). Digestibility was assessed using acid-insoluble ash as an internal marker, and relative bioavailability was estimated by linear slope-ratio regression. Data were analyzed using Zn source and level as fixed effects, blocks as random effects, and linear and quadratic polynomial contrasts to assess dose-response. Pigs fed Zn-di-tripep or ZnSO_4_ showed improved performance (*P ≤ *0.05) compared to unsupplemented pigs, particularly for average daily gain (ADG) and average daily feed intake (ADFI). From day 0 to 30, pigs fed Zn-di-tripep exhibited a quadratic response (*P ≤ *0.05) for ADG and gain-to-feed ratio (G:F), and a linear increase (*P ≤ *0.05) in final BW (FBW) and ADFI. From day 30 to 60, both sources promoted linear and quadratic increases (*P ≤ *0.05) in FBW, ADG, and G:F, and 120 mg Zn/kg diets resulted in better performance. Fecal Zn concentrations increased linearly (*P ≤ *0.05) with dietary Zn level. Pigs fed Zn-di-tripep showed a slight decrease and increase in fecal Zn (*P = *0.105) and liver Zn (*P = *0.109), respectively, and higher bone Zn concentrations (*P ≤ *0.05) than ZnSO_4_. Serum Zn increased over time (*P ≤ *0.05) in all Zn-supplemented pigs, with a trend (*P ≤ *0.10) toward higher concentrations in pigs fed Zn-di-tripep than ZnSO_4_. Apparent total tract digestibility of Zn showed linear effects (*P ≤ *0.05), with positive values only for Zn-di-tripep. In conclusion, dietary Zn–di-tripep supplementation improved Zn digestibility, serum and tissue Zn concentrations, and reduced fecal excretion compared to ZnSO_4_, suggesting greater bioavailability by allowing a reduction in Zn supplementation (∼34%), without affecting pig performance.

## Teaser

This study demonstrates that using zinc-di-tripep in pig diets improves Zn retention and reduces fecal excretion, allowing for lower dietary inclusion levels while maintaining performance.

## Introduction

Zinc (Zn) is a key trace mineral involved in several physiological processes. It contributes to acid-base regulation via carbonic anhydrase, energy metabolism through activation of glycolytic and oxidative enzymes, and supports cell proliferation, immune function, bone development, and appetite regulation (Brugger and Windisch, 2019; Skiba et al., 2022; Hansen et al., 2023; Gomes et al., 2025). Traditionally, zinc oxide (ZnO) and zinc sulfate (ZnSO_4_) are widely used in pig diets due to their availability and low cost.

However, inorganic Zn sources generally exhibit lower intestinal absorption (Dalto et al., 2019), often exacerbated by interactions with dietary phytate, calcium (Ca^2+^), and fiber (Nielsen et al., 2022), leading to increased fecal Zn excretion (Nollet et al., 2007). Given these limitations, organic chelates such as Zn-di-tripeptides have emerged as promising alternatives due to their potentially superior bioavailability (Xie et al., 2019; Ma et al., 2021; Nielsen et al., 2022). Chelation of Zn to peptides alters its physicochemical properties, enhancing its solubility and stability (Udechukwu et al., 2018). This promotes absorption via specific peptide transporters such as PepT1, while minimizing competition with other divalent cations like Ca^2+^ and iron (Nitrayová et al., 2012; Zhang et al., 2018). Moreover, organic Zn supplementation has been associated with improved apparent digestibility, intestinal absorption, tissue retention, and performance, as well as reduced fecal Zn excretion compared to inorganic salts (Nollet et al., 2007; Liu et al., 2016; Ma et al., 2021; Hu et al., 2022; Skiba et al., 2022; Soto et al., 2024).

In this context, Zn-di-tripeptide was chosen as a source of peptide-bound Zn for direct and graded comparison with ZnSO_4_ supplemented in diets for growing pigs, given reported advantages in transporter-mediated absorption and solubility, reduced antagonism with dietary compounds, and the limited dose-response evidence in pig studies. Previously, other studies assessed organic Zn sources in pig diets, for example, dietary inclusion of Zn glycinate (160 mg/kg) has been shown to increase femoral mineral concentration and density in pigs than ZnSO_4_ (160 mg/kg) (Skiba et al., 2022), while Zn methionine supplementation (10 mg/kg) improved daily weight gain, digestibility, and Zn retention relative to ZnO (100 mg/kg) (Nitrayová et al., 2012). Similarly, supplementation with Zn-protein chelates (200 and 400 mg/kg) elevated serum Zn concentrations in growing pigs compared to ZnO (Matte et al., 2017). In weaned piglets, Zn glycinate supplementation (100 mg/kg) improved G:F and Zn digestibility compared to ZnSO_4_ (100 mg/kg) (Diao et al., 2021).

Despite these benefits, the literature presents inconsistencies regarding the relative bioavailability of organic vs. inorganic Zn sources, especially ZnSO_4_, which is highly soluble and potentially well absorbed (Wedekind et al., 1994). In weaned piglets, Nielsen et al. (2022) found no significant differences in digestibility, performance, or serum Zn concentrations between 3 organic sources (Zn–glycine, Zn–amino acid chelate, and Zn–hydroxyl ligand chelate) and the inorganic sources ZnO and ZnSO_4_ (100 mg/kg). Likewise, Paulicks et al. (2011) observed similar bioavailability among Zn glycinate chelates, Zn–amino acid chelates, and ZnSO_4_ (15, 30, and 50 mg/kg) in piglet diets based on performance, plasma concentration, and Zn digestibility.

However, key gaps remain regarding Zn kinetics along the gastrointestinal tract, homeostatic regulatory mechanisms (e.g., absorption, serum peaks, excretion), and Zn retention in hepatic and bone tissues. In the present study, we hypothesized that a novel Zn-chelate (Zn-di-tripep) exhibits greater absorption and body retention than ZnSO_4_. Therefore, this study aimed to compare the apparent total tract digestibility of Zn, Zn concentrations in feces, serum, liver, and bone, and growth performance of growing pig supplemented with increasing levels of Zn-di-tripep and ZnSO_4_.

## Materials and Methods

### Ethics statement

All experimental protocols were approved by the Ethics Committee on Animal Use of the Universidade Federal de Viçosa (UFV, Brazil) (protocol no. 071/2023). All methods were reported in accordance with ARRIVE guidelines (https://arriveguidelines.org/arrive-guidelines).

### Experimental design, animals, housing, and dietary treatments

A total of 90 entire hybrid male pigs of commercial lineage (Landrace × Large White, 56 day-old, 21.61 ± 0.314 kg body weight [BW]) received diets without supplementation of mineral premix during a 7‐day adaptation period. After the 7 days, pigs (25.44 ± 0.302 kg BW) were allocated to pens (*n *= 1 pig per pen; experimental unit) based on BW (very light, light, medium, heavy, and very heavy pigs), and the pens were assigned to 1 of 9 dietary treatments (*n *= 10 replicates/dietary treatment) in a randomized complete block design following a 2 × 4 factorial in addition to a negative control (no supplemental Zn). The experiment was performed in 2 rounds of 60 days each. Each round consisted of 45 pens (*n *= 5 pigs/treatment). Each BW block (*n *= 5) consisted of 9 pigs (*n *= 1 pig from each treatment) in each of the 2 rounds.

The dietary treatments consisted of a combination of Zn sources (ZnSO_4_ vs. Zn-di-tripep) and supplemental levels (30, 60, 90, and 120 mg/kg diet) + 1 negative control. The Zn-di-tripep contained 22% of Zn (Yes, Campinas, SP, Brazil), while ZnSO_4_ contained 35% of Zn (Bauminas Agro, Itapecerica, MG, Brazil). All diets were corn and soybean meal-based with the addition of synthetic amino acids, without phytase supplementation and offered as mash. Diets and mineral-vitamin premix were formulated to meet nutritional requirements for growing pigs (Table 1), according to ­Rostagno et al. (2017).

**Table 1.** Composition of diets fed to growing male pigs during the experiment (as-fed)^1^

Ingredients (%)	Growing	
	Adaptation^3^	Control^4^
**Ground corn (22.2 mg of Zn/kg), 7.86% CP**	69.858	69.267
**Soybean meal (44.1 mg of Zn/kg), 45.4% CP**	23.646	23.736
**Soybean oil**	2.925	3.127
**Dicalcium phosphate**	1.750	1.750
**Calcitic limestone**	0.612	0.611
**Common salt**	0.449	0.449
**L-lysine HCl, 98.1%**	0.386	0.385
**DL-methionine, 99.5%**	0.154	0.155
**L-threonine, 96.8%**	0.166	0.166
**L-tryptophan, 99%**	0.031	0.031
**L-valine, 95.5%**	0.024	0.024
**Premix^2^**	–	0.300
**Calculated chemical composition**		
**Metabolizable energy, kcal/kg**	3350	3350
**Crude protein, %**	17.01	17.01
**SID^5^ lysine, %**	1.069	1.069
**SID methionine + cysteine, %**	0.631	0.631
**SID threonine, %**	0.695	0.695
**SID tryptophan, %**	0.214	0.214
**SID valine, %**	0.738	0.738
**Total Ca, %**	0.722	0.722
**Standardized total tract digestible P, %**	0.357	0.357
**Total Zn, mg/kg**	25.94	25.86
**Total Na, %**	0.190	0.190

1 The dietary treatments were supplemented with different sources and levels. The Zn values analyzed in the diets containing the Zn-di-tripep and ZnSO_4_ source at levels of 30, 60, 90, and 120, respectively, were: 55.82 mg/kg, 76.55 mg/kg, 106.95 mg/kg, 133.4 mg/kg, and 55.80 mg/kg, 78.41 mg/kg, 106.72 mg/kg, 132.24 mg/kg. The Zn-di-tripep and ZnSO_4_ sources were declared to have 22% and 35% total Zn, with the total Zn concentration analyzed being 21.5% and 36.5%, respectively.

2 Zn-free vitamin-mineral premix providing the following per kg of diet: vitamin A, 7270 IU/kg; vitamin D_3_, 1599 IU/kg; vitamin E, 43.6 IU/kg; vitamin K_3_, 3.15 mg/kg; vitamin B_1_, 1.066 mg/kg; vitamin B_2_, 3.974 mg/kg; vitamin B_6_, 2.133 mg/kg; vitamin B_12_, 0.022 mg/kg; niacin, 31.5 mg/kg; pantothenic acid, 16.0 mg/kg; folic acid, 0.339 mg/kg; biotin, 0.107 mg/kg; choline chloride, 213.3 mg/kg; Mn sulfate, 33.9 mg/kg; Fe sulfate, 67.9 mg/kg; Cu sulfate, 10.18 mg/kg; iodine, 0.838 mg/kg; sodium selenite, 0.305 mg/kg.

3 Analyzed zinc concentration = 26.15 mg/kg diet (as-fed basis).

4 Analyzed zinc concentration = 25.60 mg/kg diet (as-fed basis).

5 Standardized ileal digestible.

At the beginning of the experiment (day 0), animals were weighed and identified using numbered ear tags. The pigs were housed in a masonry and ceramic tile facility, with 2 rows (with a central aisle) of masonry floor pens (2.30 m × 2.10 m, 4.83 m^2^) equipped with front masonry trough-type feeders and central nipple drinkers. Animals were fed ad libitum (except on blood collection days) and had free access to water throughout the experiment.

Room temperature and relative humidity were recorded by a data logger (Omega, model OM-HL-SP USB IP67; São Paulo, SP, Brazil). Average temperature and relative humidity during the experiment were 22.31 ± 4.39 °C and 80.77 ± 17.28%, respectively. The growing facility was ventilated manually with the help of side curtains, a natural arborization system and a ridge vent. A 12‐h light:12‐h dark cycle was maintained.

### Growth performance

Animals were weighed on day 0, 30, and 60 of the experiment, using a digital scale (Prix, model 2098/59; São Bernardo do Campo, SP, Brazil). Offered diet and leftovers were monitored daily throughout the experiment. The average daily feed intake (ADFI, kg), the average daily BW gain (ADG, kg), and gain-to-feed ratio (G:F, kg:kg) were calculated. Results are presented as 0 to 30 (phase I), 30 to 60 (phase II), and 0 to 60 days (overall period).

### Sampling and preparation of feces and diet samples

From day 50, the animals were fed diets containing acid-insoluble ash ([AIA], Celite hyflo super cel, 10 g/kg diet) for 5 days. On day 55, feces were manually collected (from 0700 to 1900 hours, *n *= 10 animals/treatment) from the floor, using powder-free nitrile gloves. Then, the total feces collected were placed in properly labeled plastic bags, and were stored at a temperature of −20 °C until analysis.

The feces samples were then thawed at room temperature for 12 h and manually homogenized using disposable gloves in a plastic container. Homogenization quality was verified by visual inspection and by determining the coefficient of variation of Zn in samples, which was maintained below 5%. Then, 2 samples (technical duplicate) of feces and experimental diets were weighed (110 g each, totaling 220 g) separately on an analytical scale (Bel engineering, model M4102; Monza, Italy), then dried at 55 °C for 72 h (AOAC, 2019) in a forced-air oven (Tecnal, model SF-325 NM; Piracicaba, SP, Brazil). Subsequently, feces and diet samples were ground separately in a micro-powder grinding mill (Tecnal, model R-TE-350; Piracicaba, SP, Brazil) and stored in polyethylene containers.

### Calculations of zinc intake, apparent total tract digestibility of zinc and apparently digested zinc per kilogram of consumed diet

Zinc concentrations in diets and feces samples were used to calculate the total intake of Zn (in mg/d) and the fecal excretion of Zn as previously reported by Sakomura and Rostagno (2016), respectively. The apparent total tract digestibility (ATTD) of Zn was calculated using the following equation:


$$\text{ATTD of Zn}\left(\text{\%}\right)=100-\left(\left(\left(\frac{{\text{AIA}}_{\text{diet}}}{{\text{AIA}}_{\text{feces}}}\text{\%}\right)\times \left(\frac{{\text{Zn}}_{\text{feces}}}{{\text{Zn}}_{\text{diet}}}\text{\%}\right)\right)\times 100\right)$$

Digested Zn per kg of consumed diet (from day 50 to 55) was calculated using the following equation (Nielsen et al., 2022):


$${\mathrm{Digested}\;\mathrm{Zn}}_{50-55}(\frac{\mathrm{mg}}{\mathrm{kg}})=({\mathrm{total}\;\mathrm{Zn}\;\mathrm{intake}}_{50-55}\times (\frac{{{\mathrm{ATTD}\;\mathrm{of}\;\mathrm{Zn}}_{50-55}}_{}}{100}))÷{\mathrm{total}\;\mathrm{diet}\;\mathrm{intake}}_{50-55}$$

### Blood sampling and analyses

Prior to the beginning of the trial, on the last day of the adaptation period, baseline blood was collected from the animals prior to receiving the dietary treatments. On day 59, the animals were divided into 5 groups of 9 pigs for a scheduled diet fasting of 12 h. Then, on day 60, a bolus of Zn was fed with 300 g of corn meal for 10 timed min. The amount of Zn in the bolus of each treatment was calculated based on the animals’ average daily feed intake (e.g., 3.5 kg of diet/d). Blood samples were collected from all animals (*n *= 10 pigs/treatment) 5 min before the bolus (time 0, pre-bolus), and at 1 h (time 1) and 3 h (time 2) after the bolus consumption. After the last collection (3 h post-meal), all animals were fed ad libitum (from 1900 to 2200 hours on day 60) their respective dietary treatments before starting the pre-slaughter diet fasting.

Prior to blood collection, all the glass tubes used were decontaminated with 2% HCl for a period of 5 min, rinsed with deionized water and dried in an oven at 105 °C for 30 min. Blood samples (10 mL) were collected by venipuncture from the orbital sinus using 1.6 × 40 mm (16G 1 ½) hypodermic needles into glass tubes with no anticoagulant. After at least 4 h standing at room temperature, whole blood samples were centrifuged (Centrilab analog centrifuge, model 80-2B) at 1,800 × *g* for 10 min at 4 °C for the collection of serum that was then split into aliquots (Eppendorf-tubes) and stored at −20 °C until analysis. A commercial kit (Randox—São Bernardo do Campo, SP, Brazil) was used to analyze Zn concentrations (colorimetric method, Cat. ZN2341) in a Labmax automatic biochemical analyzer (Labtest, model Labmax 450i—Lagoa Santa, MG, Brazil).

### Slaughter procedures, and sampling and preparation of liver and bone samples

On day 61 of each round (0700 to 1000 hours), after 12 h of diet fasting, the animals (*n *= 10 pigs/treatment) were electrically stunned (240 volts for 3 s), followed by exsanguination, at the UFV slaughterhouse. The liver and metatarsal bones of all animals were collected at slaughter to determine Zn concentrations. Four 3 cm × 3 cm pieces were cut from each lobe of the liver. The collected fore and hind legs were deboned using tweezers and scalpels to expose the metatarsals. The left third and fourth metatarsals were cleaned to remove tissue and cartilage using scalpels and surgical scissors. The bones were boiled for 1 h in a slow cooker, and then the remaining soft tissue was removed with tweezers and a scalpel.

The liver and bones were then dried in an oven at 65 °C for 72 h. Afterwards, the bones were defatted with petroleum ether in a Soxhlet extractor (Laborchemike, model LCK-91793; Curitiba, PR, Brazil) and magnetic stirrer with heating plate (Quimis, model 0261-I2; Diadema, SP, Brazil) for 4 h. The ether was then evaporated from the bones for 30 min in an oven at 105 °C (Tecnal, model SF-325 NM; Piracicaba, SP, Brazil) ­(Detman et al., 2025). The liver and bones were ground in a closed chamber ball mill, dried in an oven at 105 °C (Tecnal, model TE-393-180L; Piracicaba, SP, Brazil) for 2 h (Lee et al., 2021) for dry matter (DM) analysis and subsequently stored in previously identified plastic flasks for Zn analysis.

### Analysis in feces, diet, liver, and bone samples

The concentrations of the AIA marker present in the diets and feces were analyzed by determining the residue insoluble in HCl using the gravimetric method (Rupolo et al., 2023).

Prior to analyzing zinc concentration in the samples, all the glass containers used were decontaminated with 2% HCl for a period of 5 min, rinsed with deionized water and dried in an oven at 105 °C for 30 min. After weighing 0.5 g of the ground material (feces, diets, livers, and bones), a total of 10 mL of the mixture of nitric acid plus perchloric acid in a ratio of 4:1 was added inside a digestion tube in an exhaust hood. The digestion tubes containing the material and the acid solution were then placed on a hot plate preheated to 80 °C until they reached a temperature of 200 °C. After the extract became crystalline, it was removed from the plate to cool, and the volume was made up to 25 mL with deionized water. Following the procedures described in AOAC (2019), DM (method no. 930.15) and Zn concentrations (method no. 999.11) were analyzed. Zinc concentrations in the feces (mg/kg DM), diet (mg/kg, as-fed basis), liver (mg/kg DM) and bones (mg/kg DM) were accurately quantified by flame atomic absorption spectrometry (Agilent Technologies, model AA240 FS, Barueri, SP, Brazil).

### Statistical procedures

Statistical analyzes were performed using the procedure of SAS (SAS, 9.4). Pig was considered as an experimental unit. A standardized residuals analysis was performed before ANOVA or ANCOVA. Outliers were set at residuals ≥ |3|, without excluding any replicate. The normality of variable errors was assessed using the Shapiro-Wilk test. The sources and levels of Zn and the diet without Zn supplementation (2 × 4 + 1) constituted the treatments and were considered fixed effects in the model. Animal, blocks, and residual error were considered random factors. Data on serum Zn concentrations were evaluated as repeated measures over time, with pig as the subject. Initial BW and baseline blood were considered as covariates for performance data and serum Zn concentrations, respectively. Treatment effects (*P ≤ *0.05) on the dependent variables were analyzed via two-way ANCOVA or ANOVA. When a significant interaction was found (*P ≤ *0.10), a partitioned analysis was carried out using the SLICE statement. The source effect was detected using the t-test. Differences between levels were compared using Tukey’s post hoc test. Differences between the Zn sources (Zn-di-tripep or ZnSO_4_) and the negative control (diet without Zn supplementation) were made using Dunnett’s test. A trend was declared when 0.05 *<* *P ≤ *0.10. Polynomial contrast was conducted to measure the linear and quadratic effects for increasing the Zn levels on all measurements, considering the negative control treatment. The data were fitted to linear slope-ratio equations only to estimate the relative bioavailability of Zn-di-tripep in relation to ZnSO_4_. Relative Zn bioavailability was calculated from regression of ADG and G:F (day 0 to 60), Zn in liver, Zn in blood (0 h), ATTD of Zn (day 50 to 55), and Zn in metatarsal bone on supplemental Zn with ZnSO_4_ set at 100% bioavailability (standard source). The formula used to compute bioavailability was: $(\frac{a}{b})\times 100$, where *a* is the slope for the Zn-di-tripep and *b* is the slope for the ZnSO_4_ (Sakomura and Rostagno, 2016). Results were reported as averages with pooled SEM.

## Results

### Growth performance

Pigs fed both Zn sources tested showed (*P ≤ *0.05) better performance results compared to those fed diets without Zn supplementation, except in phase I (day 0 to 30), where there was no difference in G:F between animals fed diets containing ZnSO_4_ vs. without Zn supplementation (Table 2).

**Table 2.** Growth performance of growing pigs fed different sources and levels of zinc

Item¹	Dietary treatments									SEM²	*P*-value^3^		
	Control^α^	Zn-di-tripep (mg/kg diet)				Zn sulfate (mg/kg diet)							
	0	30	60	90	120	30	60	90	120		Source	Level*	Inter
**Phase I (day 0 to 30)**													
**IBW, kg**	25.44	25.42	25.46	25.44	25.47	25.44	25.45	25.41	25.44	0.302	-	-	-
**FBW, kg**	57.34	60.77	61.09	59.64	61.85	59.64	60.51	59.74	61.42	0.494	0.503	0.156	0.760
**ADG, kg/d**	1.04	1.17	1.18	1.14	1.19	1.14	1.16	1.14	1.19	0.010	0.494	0.167	0.773
**ADFI, kg/d**	2.03	2.16	2.15	2.08	2.27	2.14	2.13	2.20	2.24	0.020	0.672	0.087	0.336
**G:F, kg:kg**	0.52	0.54	0.55	0.55	0.53	0.53	0.55	0.52	0.53	0.004	0.174	0.314	0.306
**Phase II (day 30 to 60)**													
**FBW, kg**	87.30	101.44	100.96	97.69	102.10	96.84	98.65	96.85	101.98	0.818	0.088	0.018	0.434
**ADG, kg/d**	1.02	1.36	1.31	1.26	1.36	1.24	1.34	1.23	1.35	0.018	0.191	0.029	0.216
**ADFI, kg/d**	2.88	3.22	3.21	3.11	3.30	3.20	3.20	3.09	3.33	0.031	0.974	0.013	0.972
**G:F, kg:kg**	0.34	0.42	0.41	0.41	0.41	0.39	0.42	0.40	0.40	0.007	0.121	0.741	0.210
**Overall period (day 0 to 60)**													
**ADG, kg/d**	1.04	1.26	1.25	1.20	1.27	1.19	1.25	1.19	1.27	0.012	0.095	0.016	0.381
**ADFI, kg/d**	2.43	2.71	2.66	2.59	2.80	2.67	2.67	2.65	2.79	0.023	0.944	0.005	0.795
**G:F, kg:kg**	0.36	0.39	0.39	0.39	0.38	0.37	0.39	0.37	0.38	0.004	0.166	0.315	0.338

* Main effect of level for ADFI, day 0 to 30 (30: 2.15^b^, 60: 2.14^b^, 90: 2.14^b^, 120: 2.25^a^, SEM = 0.070); FBW, day 30 to 60 (30: 99.14^ab^, 60: 99.93^ab^, 90: 97.27^b^, 120: 102.04^a^, SEM = 2.113); ADG, day 30 to 60 (30: 1.29^ab^, 60: 1.33^a^, 90: 1.25^b^, 120: 1.36^a^, SEM = 0.052); ADFI, day 30 to 60 (30: 3.21^ab^, 60: 3.21^ab^, 90: 3.10^b^, 120: 3.32^a^, SEM = 0.091); ADG, day 0 to 60 (30: 1.22^ab^, 60: 1.25^a^, 90: 1.19^b^, 120: 1.27^a^, SEM = 0.036); ADFI, day 0 to 60 (30: 2.69^b^, 60: 2.66^b^, 90: 2.62^b^, 120: 2.79^a^, SEM = 0.067).

α Control vs. average of all levels within Zn-di-tripep—for all variables at all studied phases *P ≤ *0.010; Control vs. average of all levels within Zn sulfate—G:F day 0 to 30 *P = *0.116, for all other variables at all studied phases *P ≤ *0.010.

1 IBW: initial body weight, FBW: final body weight, ADG: average daily gain, ADFI: average daily feed intake, G:F: gain-to-feed ratio.

2 Pooled standard error of the mean

3 Significance probability of the analysis of covariance.

In phase I (from day 0 to 30), pigs fed diets supplemented with 120 mg Zn/kg tended (*P ≤ *0.10) to show higher ADFI compared to the other Zn levels (Table 2). In phase II (from day 30 to 60), pigs receiving Zn-di-tripep diets tended (*P ≤ *0.10) to have greater FBW than those fed ZnSO_4_ source, similar to the result for ADG in the total period (from day 0 to 60). Regarding the level effect (*P ≤ *0.05), pigs fed diets supplemented with 120 mg Zn/kg had higher FBW and ADFI compared to those fed 90 mg Zn/kg. For ADG, pigs fed diets supplemented with 60 or 120 mg of Zn/kg exhibited the highest values compared to those fed 90 mg of Zn/kg. For the total period (from day 0 to 60), pigs fed diets supplemented with 60 or 120 mg Zn/kg exhibited higher ADG compared to those fed 90 mg Zn/kg, while ADFI was higher in pigs fed 120 mg Zn/kg than the other levels.

In phase I, pigs fed diets supplemented with either Zn-di-tripep or ZnSO_4_ showed significant linear improvements (*P ≤ *0.05) for FBW, ADG, and ADFI, but no linear effect was detected for G:F (Table 3). In phase II, both Zn sources promoted significant (*P ≤ *0.05) linear effect for FBW, ADG, and G:F, indicating that increasing dietary Zn levels improved these performance variables. Average daily feed intake responded (*P ≤ *0.05) linearly to increasing Zn levels. Over the total period, significant linear (*P ≤ *0.05) effect was observed for ADG and G:F across both Zn sources. Average daily feed intake showed (*P ≤ *0.05) only linear effects.

**Table 3.** Linear and quadratic contrasts for growth performance of growing pigs fed different sources and levels of zinc

Item	Sources	Polynomial contrasts	*P*-value
**Phase I (day 0 to 30)**			
**FBW, kg**	Zn-di-tripep	Linear	0.001
		Quadratic	0.132
	Zn sulfate	Linear	0.001
		Quadratic	0.306
**ADG, kg/d**	Zn-di-tripep	Linear	0.002
		Quadratic	0.037
	Zn sulfate	Linear	0.001
		Quadratic	0.186
**ADFI, kg/d**	Zn-di-tripep	Linear	0.015
		Quadratic	0.793
	Zn sulfate	Linear	0.003
		Quadratic	0.720
**G:F, kg:kg**	Zn-di-tripep	Linear	0.395
		Quadratic	0.004
	Zn sulfate	Linear	0.403
		Quadratic	0.251
**Phase II (day 30 to 60)**			
**FBW, kg**	Zn-di-tripep	Linear	0.001
		Quadratic	0.001
	Zn sulfate	Linear	0.001
		Quadratic	0.046
**ADG, kg/d**	Zn-di-tripep	Linear	0.001
		Quadratic	0.003
	Zn sulfate	Linear	0.001
		Quadratic	0.009
**ADFI, kg/d**	Zn-di-tripep	Linear	0.004
		Quadratic	0.184
	Zn sulfate	Linear	0.002
		Quadratic	0.346
**G:F, kg:kg**	Zn-di-tripep	Linear	0.001
		Quadratic	0.002
	Zn sulfate	Linear	0.001
		Quadratic	0.003
**Overall period (day 0 to 60)**			
**ADG, kg/d**	Zn-di-tripep	Linear	0.001
		Quadratic	0.002
	Zn sulfate	Linear	0.001
		Quadratic	0.018
**ADFI, kg/d**	Zn-di-tripep	Linear	0.001
		Quadratic	0.394
	Zn sulfate	Linear	0.001
		Quadratic	0.308
**G:F, kg:kg**	Zn-di-tripep	Linear	0.056
		Quadratic	0.001
	Zn sulfate	Linear	0.007
		Quadratic	0.100

The quadratic contrast for ADG consistently indicated (*P ≤ *0.05) a downward concave response, i.e., ADG increased with Zn supplementation up to an intermediate point and then stabilized or decreased, across all phases (except in phase I for the ZnSO_4_) (Table 3). On the other hand, G:F exhibited (*P ≤ *0.05) a downward-curved response pattern across all phases for Zn-di-tripep, improving up to a peak and then declining at higher inclusions. Zinc sulfate responded similarly, but with no effects in phase I and a trend (*P ≤ *0.10) in the total period.

### Zinc intake, apparent total tract digestibility of zinc, and apparently digested zinc per kg of consumed diet

From day 30 to 60 and 0 to 60, there was an interaction trend (*P ≤ *0.10) for Zn intake (Table 4). When unfolding this interaction effect, pigs fed 120 mg/kg of both sources had the highest Zn intakes, and those fed 90 mg/kg of Zn-di-tripep/kg had higher intakes than the other levels of the sources. Independently of Zn source, pigs supplemented with 30 mg/kg had the lowest Zn intakes.

**Table 4.** Zinc (Zn) intake (in mg/d) during the experimental phases, apparent total tract digestibility (ATTD) of Zn, and apparently digested (AD) Zn per kg of consumed diet (as-fed basis) in growing pigs fed different sources and levels of Zn

Item	Dietary treatments									SEM¹	*P*-value²		
	Control	Zn-di-tripep (mg/kg diet)				Zn sulfate (mg/kg diet)							
	0	30	60	90	120	30	60	90	120		Source	Level*	Inter
**Zn intake (day 0 to 30)**	49.85	121.09	165.01	235.41	303.66	112.76	172.16	235.03	290.22	8.672	0.306	<0.001	0.262
**Zn intake (day 30 to 60)**	72.09	179.96^e^	245.95^d^	352.18^b^	440.66^a^	168.69^e^	258.52^d^	329.37^c^	431.35^a^	12.657	0.082	<0.001	0.074
**Zn intake (day 0 to 60)**	60.03	151.65^d^	204.18^c^	293.79^b^	373.49^a^	140.73^d^	215.34^c^	282.20^b^	360.79^a^	10.627	0.091	<0.001	0.074
**Zn ATTD, % (day 50 to 55)**	−11.06	0.09	1.90	3.64	11.55	−4.81	−6.91	−1.84	−1.34	1.506	0.001	0.105	0.554
**AD Zn, mg/kg (day 50 to 55)**	−3.11	0.22	3.11	4.11	15.41	−2.53	−2.49	−1.97	−1.70	1.387	<0.001	0.113	0.188

* Level effect—Zn intake, day 0 to 30 (30: 116.92^d^, 60: 168.58^c^, 90: 235.21^b^, 120: 296.19^a^, SEM = 7.697); Zn intake, day 30 to 60 (30: 174.03^d^, 60: 252.56^c^, 90: 340.77^b^, 120: 436.00^a^, SEM = 9.353); Zn intake, day 0 to 60 (30: 146.19^d^, 60: 209.76^c^, 90: 287.99^b^, 120: 367.13^a^, SEM = 7.400); main effect for ATTD of Zn, day 50 to 55 (30: −2.36^b^, 60: −1.72^ab^, 90: 0.89^ab^, 120: 5.10^a^, SEM = 4.956).

a-e Interaction effect.

α Control vs. average of all levels within Zn-di-tripep—for all variables at all studied phases *P < *0.001; Control vs. average of all levels within Zn sulfate—ATTD of Zn day 50 to 55 *P = *0.223 and Apparently digested Zn day 50 to 55 *P = *0.391, for all other variables at all studied phases *P < *0.001.

1 Pooled standard error of the mean.

2 Significance probability of the analysis of variance.

The pigs fed both Zn sources showed (*P ≤ *0.05) higher Zn consumption during all the experimental phases evaluated than the pigs fed diets without Zn supplementation (Table 4). In addition, Zn intake tended (*P ≤ *0.10) to be higher in pigs fed Zn-di-tripep compared to those fed ZnSO_4_ from day 30 to 60 and 0 to 60. In both phases evaluated, regardless of the Zn source, Zn intake increased linearly (*P ≤ *0.05) in response to the increase in Zn supplementation in the diets (Table 5).

**Table 5.** Linear and quadratic contrasts for zinc (Zn) intake during the experimental phases, apparent total tract digestibility (ATTD) of Zn, and apparently digested Zn per kg of consumed diet in growing pigs fed different sources and levels of zinc

Item	Sources	Polynomial contrasts	*P*-value
**Zn intake (day 0 to 30), mg/d**	Zn-di-tripep	Linear	0.001
		Quadratic	0.603
	Zn sulfate	Linear	<0.001
		Quadratic	0.216
**Zn intake (day 30 to 60), mg/d**	Zn-di-tripep	Linear	0.001
		Quadratic	0.979
	Zn sulfate	Linear	<0.001
		Quadratic	0.846
**Zn intake (day 0 to 60), mg/d**	Zn-di-tripep	Linear	0.001
		Quadratic	0.800
	Zn sulfate	Linear	<0.001
		Quadratic	0.540
**ATTD of Zn, % (day 50 to 55)**	Zn-di-tripep	Linear	0.014
		Quadratic	0.665
	Zn sulfate	Linear	0.044
		Quadratic	0.684
**Apparently digested Zn, mg/kg diet (day 50 to 55)**	Zn-di-tripep	Linear	0.024
		Quadratic	0.294
	Zn sulfate	Linear	0.005
		Quadratic	0.650

From day 50 to 55, pigs fed diets supplemented with Zn-di-tripep exhibited (*P ≤ *0.05) higher Zn ATTD and apparently digested Zn compared to those fed ZnSO_4_ or without Zn supplementation. For all dietary treatments where ZnSO_4_ was supplemented, the ATTD of Zn was negative. The amounts of apparently digested Zn per kg of consumed diet were greater than zero when Zn-di-tripep was supplemented to the diets. A level trend was observed (*P ≤ *0.10) for Zn ATTD, in which pigs fed 120 mg Zn/kg exhibited higher values compared to those fed 30 mg Zn/kg, but did not differ from the other levels (Table 4). A significant linear effect (*P ≤ *0.05) was observed for Zn ATTD and apparently digested Zn, with increasing Zn levels resulting in higher values for both sources (Table 5), rather than a second-degree polynomial.

### Serum zinc concentrations

Serum Zn concentration at the end of the adaptation period before the pigs received the dietary treatments was 52.96 ± 2.45 mcg/dL. The pigs fed both Zn sources exhibited (*P ≤ *0.05) higher serum Zn concentrations than the pigs fed diets without Zn supplementation at the different times evaluated (Table 6). Pigs fed Zn-di-tripep showed a trend toward (*P ≤ *0.10) higher serum Zn concentrations compared to those fed ZnSO_4_ at all evaluated times, with percentage increases of 6.6%, 5.5%, and 5.1% at 0, 1, and 3 h, respectively. At time 0 h, pigs fed diets supplemented with 30 mg of Zn/kg showed (*P ≤ *0.05) the lowest serum Zn concentrations. At time 1 h and 3 h, pigs fed diets supplemented with 120 mg Zn/kg had (*P ≤ *0.05) higher Zn concentrations compared to those fed 30 or 90 mg Zn/kg. In addition, pigs fed diets supplemented with 30 mg of Zn/kg showed (*P ≤ *0.05) lower values at time 3 h than those fed 60 mg of Zn/kg.

**Table 6.** Serum zinc concentrations (mcg/dL) in growing pigs fed different sources and levels of zinc at 3 times after bolus feeding (on day 60)

Item^1^	Dietary treatments									SEM²	*P*-value^3^		
	Control^α^	Zn-di-tripep (mg/kg diet)				Zn sulfate (mg/kg diet)							
	0	30	60	90	120	30	60	90	120		Source	Level*	Inter
**0 h**	25.37	76.77	92.01	99.18	102.29	75.36	86.55	90.06	95.30	2.734	0.078	0.001	0.672
**1 h**	25.44	110.54	116.87	107.92	122.06	101.71	111.33	106.85	113.67	2.247	0.068	0.036	0.905
**3 h**	25.77	112.90	116.68	110.46	123.79	98.16	112.47	110.70	119.83	2.428	0.070	0.002	0.503

* Level effect—Zn concentrations at 0 h (30: 74.34^b^, 60: 89.13^a^, 90: 92.83^a^, 120: 98.97^a^, SEM = 7.036); Zn concentrations at 1 h (30: 106.12^b^, 60: 114.10^ab^, 90: 107.35^b^, 120: 117.86^a^, SEM = 6.810); Zn concentrations at 3 h (30: 105.09^c^, 60: 114.46^ab^, 90: 110.57^bc^, 120: 121.81^a^, SEM = 6.157).

α Control vs. average of all levels within Zn-di-tripep or Zn sulfate—for all times *P < *0.001.

1 0 h: after 12 h of fasting (baseline), 1 h: after Zn bolus supply (except in animals without Zn supplementation), 3 h: after Zn bolus supply (except in animals without Zn supplementation).

2 Pooled standard error of the mean.

3 Significance probability of the analysis of covariance.

Serum Zn concentrations increased linearly (*P ≤ *0.05) in response to increased dietary Zn levels for both sources (Table 7). Quadratic contrasts at 0 h, 1 h, and 3 h uniformly supported (*P ≤ *0.05) a downward concave pattern, i.e., concentrations increased with increasing Zn to an intermediate point and then decreased or stabilized. This response from the quadratic models at each time point reinforced the interpretation of an increase followed by no additional benefit (or slight decrease) beyond the optimal level for both sources.

**Table 7.** Linear and quadratic contrasts for serum zinc concentrations in growing pigs fed different sources and levels of zinc

Item	Sources	Polynomial contrasts	*P*-value
**0 h**	Zn-di-tripep	Linear	0.001
		Quadratic	0.001
	Zn sulfate	Linear	0.001
		Quadratic	0.001
**1 h**	Zn-di-tripep	Linear	0.001
		Quadratic	0.001
	Zn sulfate	Linear	0.001
		Quadratic	0.001
**3 h**	Zn-di-tripep	Linear	0.001
		Quadratic	0.001
	Zn sulfate	Linear	0.001
		Quadratic	0.001

### Zinc concentrations in feces, liver, and metatarsal bones

The pigs fed both Zn sources exhibited (*P ≤ *0.05) higher concentrations of Zn in the feces, liver and bones than the animals fed diets without Zn supplementation (Table 8). Comparing the effect of Zn source, pigs receiving Zn-di-tripep had (*P ≤ *0.05) a higher Zn concentration in the metatarsal bone (percentage increase of 7.1%), while showed a slight decrease and increase in fecal Zn (*P = *0.105, percentage decrease of 4.5%) and liver Zn (*P = *0.109, percentage increase of 7.6%), respectively, compared to pig fed diets containing ZnSO_4_. Pigs fed diets supplemented with 120 mg Zn/kg showed higher Zn in liver compared to those fed 30 or 60 mg Zn/kg, while pigs fed diets supplemented with 30 mg of Zn/kg had the lowest values. The Zn concentration in bones was higher in pigs fed diets supplemented with 120 mg Zn/kg than the other levels, while pigs fed diets supplemented with 60 or 90 mg of Zn/kg showed higher values compared to those fed 30 mg of Zn/kg.

**Table 8.** Zinc concentrations in feces (on day 55), liver (on day 61), and metatarsal bones (on day 61) of growing pigs fed different sources and levels of zinc

Item	Dietary treatments									SEM¹	*P*-value²		
	Control^α^	Zn-di-tripep (mg/kg diet)				Zn sulfate (mg/kg diet)							
	0	30	60	90	120	30	60	90	120		Source	Level*	Inter
**Zn in feces, mg/kg DM**	162.07	312.52	499.72	663.90	770.36	317.00	515.45	683.25	810.00	24.741	0.105	<0.001	0.893
**Zn in liver, mg/kg DM**	69.14	113.50	147.37	167.50	175.22	114.42	140.40	148.98	167.61	4.728	0.109	<0.001	0.774
**Zn in metatarsal, mg/kg DM**	22.18	93.06	110.92	116.00	126.52	84.70	102.95	109.65	119.51	3.472	0.005	<0.001	0.991

* Level effect—Zn in feces (30: 314.76^d^, 60: 507.59^c^, 90: 673.58^b^, 120: 791.22^a^, SEM = 27.012); Zn in liver (30: 114.01^c^, 60: 143.88^b^, 90: 158.72^ab^, 120: 171.61^a^, SEM = 13.035); Zn in metatarsal (30: 88.66^c^, 60: 106.72^b^, 90: 112.64^b^, 120: 123.40^a^, SEM = 5.537).

α Control vs. average of all levels within Zn-di-tripep or Zn sulfate—for all variables *P < *0.001.

1 Pooled standard error of the mean

2 Significance probability of the analysis of variance.

Fecal, hepatic and bone Zn concentrations increased (*P ≤ *0.05) linearly as Zn supplementation increased in the diets for both sources (Table 9). The quadratic contrast for Zn in the liver showed (*P ≤ *0.05) a downward concave response for both sources, with concentrations rising toward a peak and then stabilizing. Metatarsal Zn also followed (*P ≤ *0.05) a downward concave response for both sources, increasing toward a mid-range peak and then stabilizing.

**Table 9.** Linear and quadratic contrasts for zinc concentrations in feces, liver, and metatarsal bones of growing pigs fed different sources and levels of zinc

Item	Sources	Polynomial contrasts	*P*-value
**Zn in feces, mg/kg DM**	Zn-di-tripep	Linear	0.001
		Quadratic	0.108
	Zn sulfate	Linear	0.001
		Quadratic	0.208
**Zn in liver, mg/kg DM**	Zn-di-tripep	Linear	0.008
		Quadratic	0.001
	Zn sulfate	Linear	0.009
		Quadratic	0.029
**Zn in metatarsal, mg/kg DM**	Zn-di-tripep	Linear	0.001
		Quadratic	0.001
	Zn sulfate	Linear	0.001
		Quadratic	0.001

### Relative bioavailability

Based on the criteria evaluated in this study, slope-ratio equations were considered valid when the linear coefficient was significant (*P < *0.10). The estimated average relative bioavailability of Zn-di-tripep in relation to ZnSO_4_ was 134%, that is, 100 mg of ZnSO_4_/kg could be replaced by 74.62 mg of Zn-di-tripep/kg of diet, on a Zn basis (Table 10). Since Zn ATTD included negative-very low values in the ZnSO_4_ group, we also reported bioavailability in non-ATTD variables (performance, serum, liver, bone), obtaining a relative bioavailability of 117%. Both estimates consistently indicate that Zn-di-tripep is more bioavailable than ZnSO_4_.

**Table 10.** Relative bioavailability values of linear slope-ratio equations for zinc-di-tripeptide compared to zinc sulfate fed to growing pigs^1^

Item	Source	Bioavailability (%)^2^ ^,^ ^3^
**Growth performance (day 0 to 60)**		
** ADG, kg/d**	Zn-di-tripep	115
** G:F, kg:kg**	Zn-di-tripep	136
**Zn in liver, mg/kg DM**	Zn-di-tripep	115
**Zn in blood (0 h), mcg/dL**	Zn-di-tripep	114
**ATTD of Zn, % (day 50 to 55)**	Zn-di-tripep	219
**Zn in metatarsal bone, mg/kg DM**	Zn-di-tripep	105

1 For calculations, the bioavailability of ZnSO_4_ was assumed to be 100%.

2 ADG, kg/d (day 0 to 60) = 0.0015×Zn-di-tripep + 1.1293, CI 95% for the slope = [−0.000611; 0.003611]; G:F, kg:kg (day 0 to 60) = 0.00019×Zn-di-tripep + 0.3707, CI 95% for the slope = [−0.000173; 0.000553]; Zn in liver, mg/kg DM = 0.8872×Zn-di-tripep + 81.3140, CI 95% for the slope = [0.4365; 1.3379]; Zn in blood (0 h), mcg/dL = 0.5875×Zn-di-tripep + 43.8740, CI 95% for the slope = [0.0085; 1.1665]; ATTD of Zn, % (day 50 to 55) = 0.1626×Zn-di-tripep - 8.5300, CI 95% for the slope = [0.0614; 0.2638]; Zn in metatarsal bone, mg/kg DM = 0.7721×Zn-di-tripep + 47.4120, CI 95% for the slope = [−0.0077; 1.5519].

3 ADG, kg/d (day 0 to 60) = 0.0013×ZnSO_4_ + 1.0989, CI 95% for the slope = [−0.001537; 0.004137]; G:F, kg:kg (day 0 to 60) = 0.00014×ZnSO_4_ + 0.3605, CI 95% for the slope = [−0.000364; 0.000644]; Zn in liver, mg/kg DM = 0.7717×ZnSO_4_ + 81.8100, CI 95% for the slope = [0.3597; 1.1837]; Zn in blood (0 h), mcg/dL = 0.5152×ZnSO_4_ + 43.6160, CI 95% for the slope = [−0.0479; 1.0783]; ATTD of Zn, % (day 50 to 55) = 0.0747×ZnSO_4_ − 9.6740, CI 95% for the slope = [0.0039; 0.1455]; Zn in metatarsal bone, mg/kg DM = 0.7320×ZnSO_4_ + 43.8760, CI 95% for the slope = [0.0597; 1.4043].

## Discussion

This study hypothesized that Zn-di-tripep would improve intestinal Zn absorption compared to ZnSO_4_, as was evidenced by improved Zn digestibility and a slight decrease in fecal Zn. Furthermore, greater Zn concentrations in serum and tissues would indicate superior bioavailability in growing pigs. The results obtained in the current study support this hypothesis, in which higher serum and tissue Zn concentrations are consistent with greater intestinal absorption and/or tissue retention, rather than a reduction in Zn loss via urine, because urinary Zn represents a minor loss pathway in pigs compared with fecal Zn (Poulsen and Larsen, 1995). Functionally, bone reflects medium- to long-term Zn retention (e.g., osteoblastic deposition, metalloenzyme incorporation) through deposition and incorporation into bone matrix components (Wu, 2017), while the liver reflects Zn metabolism and storage (Dalto et al., 2023). Mechanistically, peptide-bound Zn can increase effective absorption (Nitrayová et al., 2012), thereby supporting higher tissue retention at a given supplemental level. Importantly, as observed in our study, status outcomes (serum, liver, bone) may increase even when ATTD is reduced by endogenous fecal Zn under marginal supply because ATTD is influenced by unavoidable endogenous losses and does not account for Zn retained in tissues (Brugger et al., 2014; Brugger and Windisch, 2019; Nielsen et al., 2022).

In the present study, pigs fed unsupplemented diets (e.g., 25.60 mg of Zn/kg diet) showed impaired growth performance, indicating that the Zn naturally present in corn-soybean meal is insufficient to support adequate performance in growing pigs. Our findings align with those of Brugger and Windisch (2016), who evaluated Zn-deficient diets (e.g., 28.1 mg of Zn/kg diet, with feed intake limited to 450 g/d) for post-weaning piglets and reported reduced nutrient digestibility and performance. Conversely, when Zn supplementation meets requirements, several studies report limited effects on growth performance despite increases in tissue Zn. In fact, Ma et al. (2021) reported no changes in ADG, despite increased hepatic Zn concentrations. Additionally, Nitrayová et al. (2012) and Nielsen et al. (2022) reported no significant differences in growth performance between different Zn sources. This suggests that, after meeting the cellular demands for Zn to support growth, additional Zn is stored in body tissues without further improvements in performance, potentially contributing to metabolic reserves or functions beyond growth (Brugger and Windisch, 2016).

Zhang and Guo (2008) reported increased plasma growth hormone concentrations and improved ADG and G:F in weaned piglets supplemented with 200 mg/kg tetrabasic zinc chloride. However, these effects were observed during the immediate post-weaning phase, when nutritional demands and physiological responses differ markedly from those of growing pigs. In our study, the significant effects on performance indicators in pigs supplemented with 120 mg Zn/kg diet suggests that higher Zn levels may still exert additional biological effects in growing pigs, potentially through modulation of orexigenic peptides (Ohinata et al., 2009) or stimulation of Zn-dependent digestive enzymes (Brugger and Windisch, 2016). This dose–response pattern aligns with zinc homeostasis in pigs. As dietary Zn approaches the requirement, intestinal uptake supports linear improvements, but further increases are limited by homeostatic down-regulation of transport and metallothionein-mediated sequestration, yielding concave-down quadratic responses at the upper range (Brugger et al., 2014; Brugger and Windisch, 2019). Thus, linear effects reflect correction of marginal Zn supply at intermediate dietary level (in line with the significant difference vs. the control treatment and the phase-specific improvements observed), whereas the quadratic effect indicates a physiological plateau once requirements are met, with additional Zn increasingly retained in body tissues (e.g., liver, bone) rather than additional performance (Poulsen and Larsen, 1995; Brugger et al., 2014).

Collectively, our findings of serum, liver, and bone Zn concentrations were within the expected physiological range reported for pigs fed Zn supplementation (Revy et al., 2002; Matte et al., 2017; Ma et al., 2021; Nielsen et al., 2022; Hansen et al., 2023). Moderate increases in serum Zn with dietary level and source confirm that this pool is under tight homeostatic regulation (Nielsen et al., 2022; Hansen et al., 2023). Interestingly, the unexpected dip in serum Zn concentration (as observed at the 90 mg Zn/kg level) is consistent with a homeostatic regulation near the threshold from adequate to supra-nutritional intake. At this range, ZIP4 downregulation coupled ZnT1 upregulation limits Zn entry into circulation (Yu et al., 2008), while metallothionein-mediated sequestration in enterocytes promotes mucosal retention (Martínez et al., 2004; Martin et al., 2013; Holodova et al., 2019). Postprandial kinetic differences, particularly for Zn-di-tripep absorbed via PepT1 (Leibach and Ganapathy, 1996), and transient redistribution to hepatic and intestinal storage pools may also temporarily lower circulating Zn concentrations. The parallel increase in tissue Zn concentrations supports this hypothesis, suggesting redistribution and short-term storage of Zn in other pools. These mechanisms explain a temporary serum decrease at 90 mg Zn/kg, consistent with the observed concave-down pattern.

Regarding Zn ATTD, some dietary Zn levels were near the recommended requirements for growing pigs (Rostagno et al., 2017, 2024), but Zn ATTD varied according to the source, with more negative values in the ZnSO_4_ group, findings that agree with reports of negative or near-zero ATTD for ZnO and ZnSO_4_ at 100 mg Zn/kg in nursery pigs (Nielsen et al., 2022). Negative values of Zn ATTD are physiologically and methodologically plausible in trace-mineral (e.g., Zn) studies. Because absolute dietary Zn inputs are low, endogenous fecal Zn (inevitable losses of pancreatic, biliary and mucosal origin) (Brugger and Windisch, 2019) plus unabsorbed dietary Zn can, under specific conditions, exceed the estimated Zn intake due to intrinsic factors in the pigs (Nielsen et al., 2022). This reflects the contribution of endogenous Zn to feces when supply is marginal (Brugger et al., 2014).

In our data, improved ATTD of Zn and digested Zn per kg of diet for Zn-di-tripep suggests higher Zn solubility and utilization in the intestinal lumen, consistent with Nollet et al. (2007) and Schlegel et al. (2010). Zinc acts as a cofactor for carbonic anhydrase (Kivelä et al., 2005) and enzymes such as peptidase and amylase (Brugger and Windisch, 2016, 2019), which participates in nutrient digestion. Thus, adequate Zn supply supports their activity. Although improved digestive function may indirectly contribute to a favorable luminal environment (e.g., pH, proteolysis, solubility/stability of Zn forms) (Schlegel et al., 2010; Pieper et al., 2015), there is no direct increase in zinc absorption facilitated by these enzymes. Therefore, the primary mechanism explanation for source differences in Zn ATTD is chemical-physical and transport-related (e.g., lower antagonism, Zn in soluble forms, and use of peptide transport pathways) (Leibach and Ganapathy, 1996; Nitrayová et al., 2012). Consistently, slightly lower fecal Zn with Zn-di-tripep supports enhanced absorption, as previously demonstrated by Nollet et al. (2007).

It is well established that Zn absorption is dose-dependent (Poulsen and Larsen, 1995). Although intestinal homeostatic mechanisms downregulate the expression of active Zn transporters (e.g., ZIP4) as dietary Zn increase (Yu et al., 2008), this downregulation does not necessarily result in a proportional reduction in Zn absorption. This is because passive absorption pathways (e.g., paracellular transport), driven by concentration gradients, are not subject to homeostatic control. As a result, even with reduced efficiency of active transport, absolute Zn absorption can increase with higher dietary Zn levels (Paulicks et al., 2011; Dalto et al., 2023), but it is also relevant at lower supplementation levels, suggesting that passive transport mechanisms may contribute to Zn absorption. Together, these evidences support our results, in which performance, serum, liver and bone Zn showed linear and quadratic responses, fecal Zn increased proportionally to dietary Zn, and the peptide-bound source resulted higher (positive) ATTD than ZnSO_4_ at equivalent supplementation levels.

Zn-di-tripeptide is primarily absorbed via PepT1 or amino acid transporters (Leibach and Ganapathy, 1996), which are not regulated by Zn concentration in the intestinal lumen (Adibi, 1997). Therefore, chelated Zn is not present as free Zn^2+^, reducing its reactivity and preventing precipitation with dietary antagonists (Nielsen et al., 2022), favoring Zn uptake into enterocytes. In contrast, inorganic Zn salts such as ZnSO_4_ release free Zn^2+^, which competes with other divalent cations (e.g., iron and copper), forms insoluble complexes with Ca^2+^ and phytates (Nitrayová et al., 2012), and competes for passive transport channels (Martin et al., 2013). Schlegel et al. (2010) reported 22% lower solubility of ZnSO_4_ vs. Zn-glycine in piglets, reinforcing our observation of reduced ATTD of Zn with inorganic sources. As observed in our study in target tissues, these distinct mechanisms reduce interaction with limiting dietary factors and enable faster access of organic Zn to the bloodstream (Udechukwu et al., 2016), resulting in higher and sustained blood Zn concentrations (Schlegel et al., 2010; Udechukwu et al., 2016) and promoting greater tissue deposition (Schlegel and Windisch, 2006), even at lower Zn inclusion levels.

The higher bioavailability of organic Zn allows for diet formulation with lower Zn supplementation levels without compromising pig performance. Therefore, the specific absorption pathway of organic Zn sources explains their superior bioavailability, as reported by Star et al. (2012). Our findings are consistent with the literature showing that estimates of relative bioavailability vary depending on the parameter assessed (Byrne and Murphy, 2022), with serum Zn reflecting short-term flux, whereas tissue-based or retention measurements integrate cumulative Zn status in pigs. For example, tetrabasic zinc chloride consistently outperformed Zn oxide when relative bioavailability was estimated (e.g., 125% to 159%) from various parameters (e.g., plasma, liver, kidney, and bone) (Zhang and Guo, 2007), while Ma et al. (2021) found relative bioavailability values of 110.9% in the liver when evaluating chitosan–Zn compared to ZnSO_4_ for piglets. Similarly, Zn peptide/chelate sources show parameter-dependent bioavailability; for example, Zn-lysine had variable relative bioavailability when assessed by serum (e.g., 79% to 110%) (Hahn and Baker, 1993; Wedekind et al., 1994), but substantially different values when calculated from specific bones (e.g., metacarpal 60%, coccygeal vertebrae 84%) in pigs with Zn-methionine (Wedekind et al., 1994). These patterns support the interpretation of our relative bioavailability result, which is consistent with compilations of relative bioavailability in pig experiments. This demonstrates that different response criteria can result in discrepant relative bioavailability values, also associated with the level of Zn supplemented in the diet, the dietary matrix, the Zn source used as a standard (reference), the age of the pigs, the sampling timing, and the method of calculating bioavailability.

## Conclusions

The findings of this study demonstrate the superior bioavailability and environmental benefits of Zn-di-tripep compared to ZnSO_4_ supplementation in growing pig diets. Although performance was not significantly affected by Zn source, Zn-di-tripep improved Zn digestibility and tissue retention. Because of the higher serum concentrations and slightly lower fecal concentrations of Zn, Zn-di-tripep had a relative bioavailability of 134% compared to ZnSO_4_, allowing the use of lower supplementation levels to achieve similar serum and tissue Zn.

## Abbreviations:

ADFI: average daily feed intake
ADG: average daily gain
AIA: acid-insoluble ash
ANCOVA: analysis of covariance
AOAC: association of official ­analytical chemists
ATTD: apparent total tract digestibility
BW: body weight
Ca^2+^: calcium ion
DM: dry matter
FBW: final body weight
G:F: gain-to-feed ratio
PepT1: peptide transporter 1
ZIP4: zrt/Irt-like protein transporter 4
ZIP5: zrt/Irt-like protein transporter 5
ZIP10: zrt/Irt-like protein transporter 10
Zn: zinc
Zn^2+^: zinc ion
Zn-di-tripep: zinc bound to di-tripeptide carrier
ZnO: zinc oxide
ZnSO_4_: zinc sulfate
ZnT: zinc transporter
